# Mir-184 Contributes to Brain Injury Through Targeting PPAP2B Following Ischemic Stroke in Male Rats

**DOI:** 10.3389/fnmol.2021.613887

**Published:** 2021-03-23

**Authors:** Huajun Yang, Yifan Zhang, Hongqun Chen, Yingwu Zhu, Yuan Li, Fu Ouyang, Lan Chu, Daishun Liu

**Affiliations:** ^1^Department of Neurology, Affiliated Hospital of Guizhou Medical University, Guizhou Medical University, Guiyang, China; ^2^Department of Respiratory Medicine, The First People’s Hospital of Zunyi, The Third Affiliated Hospital of Zunyi Medical University, Zunyi, China

**Keywords:** ischemia stroke, mir-184, brain injury, apoptosis, type 2 phosphatidic acid phospatase gene B

## Abstract

Our previous study revealed that miR-184 expression is significantly altered in the brain following ischemic stroke in rats. However, it is unknown whether this alteration in miR-184 expression contributes to brain injury after ischemic stroke. Here, we aim to address the potential of miR-184 to impact nerve injury following ischemia and reperfusion. Rats received ICV injection of miR-184 adenovirus or empty vector and were subjected to right middle cerebral artery occlusion (MCAO) to establish an ischemic stroke model. We cultured SH-SY5Y cells under oxygen-glucose deprivation/reoxygenation (OGD/R) and transfected them with miR-184 lentivirus to explore the primary mechanisms. To evaluate miR-184 expression, neurological function deficits, the cerebral infarct volume, cell viability, and apoptosis, qRT-PCR analysis of miR-184 expression, the modified neurological severity score (mNSS) system, TTC staining, the CCK-8 assay, flow cytometry, and dual-luciferase reporter assays were utilized. We found that miR-184 expression was downregulated and that the cerebral infarct volume and mNSSs were increased following ischemic stroke; however, increasing the level of miR-184 alleviated brain damage. Overexpression of miR-184 resulted in increased viability and reduced apoptosis of SH-SY5Y cells following OGD/R *in vitro*. We identified the phosphatidic acid phosphatase type 2B (PPAP2B) gene as a direct target gene of miR-184. In summary, our results reveal that attenuation of miR-184 levels in ischemic stroke contributes to ischemic injury through targeting PPAP2B mRNA-mediated apoptosis, which may be a promising therapeutic target for ischemic stroke.

## Introduction

As the most common type of stroke, ischemic stroke results from cerebral blood flow thrombosis or embolism, leading to oxygen and glucose deprivation, subsequent brain damage, and neurologic deficits (Han et al., 2014). Additionally, postischemic blood reperfusion further aggravates nerve injury (Kasdorf and Perlman, 2013). Decades of research have revealed much of the pathophysiology of cerebral ischemia and reperfusion injury. The process involves various elements, such as energy failure, oxidative stress, excitotoxicity, and ion imbalance, all of which can lead to a complex series of cellular reactions, changes in the expression patterns of genes, and subsequent brain damage and dysfunction in the ischemic brain (Cheon et al., 2018). There are currently limited treatment options for ischemic stroke, partly due to a lack of complete understanding of the molecular mechanism responsible for neuronal damage following ischemic stroke (Khoshnam et al., 2017a).

MicroRNAs are a family of small non-coding RNAs that negatively regulate target genes at the posttranscriptional level through hybridizing to complementary sequences located in the 3’-untranslated regions of target mRNAs. It is now evident that microRNAs are region-specific. In the brains of mammals, some microRNAs are highly expressed and implicated in neurological physiological processes and diseases (Yin et al., 2015). In particular, evidence has shown that the expression levels of many microRNAs are significantly altered after ischemic stroke in the rodent and human brain (Rink and Khanna, 2011). MicroRNAs that are expressed after stroke are involved in excitotoxicity, oxidative stress, inflammatory reactions, blood-brain barrier disruption, edema after stroke, and neuronal apoptosis (Li et al., 2018). MicroRNAs have emerged as essential regulators of gene expression in the pathology of ischemic stroke (Li et al., 2018). Our previous study found that miR-184 is among the top downregulated microRNAs in the blood of patients following ischemic stroke. The expression of miR-184 is significantly altered in the brain following ischemic stroke in rats (Wang et al., 2020). MiR-184, a highly enriched microRNA in the mammalian brain, has been shown to promote oligodendrocyte differentiation and control the proliferation and differentiation of adult neural stem/progenitor cells (Liu et al., 2010; Afrang et al., 2019). It also contributes to cardiomyocyte injury during myocardial infarction (Zou et al., 2020) and cellular apoptosis in retinoblastoma (He et al., 2019). However, it is unknown whether the alteration of miR-184 expression contributes to brain injury after ischemic stroke. The present study investigation aims to address the potential of miR-184 to impact nerve injury following ischemia and reperfusion *in vivo* and *in vitro*. The results may shed new light on the underlying mechanism of poststroke brain injury and provide clues for new gene therapies for stroke recovery.

## Materials and Methods

### Animals and Protocols

Male Sprague-Dawley rats weighing 250–300 g were obtained from Tianqin Biotech Co. (Changsha, China), housed in individual cages at 20 ± 3°C and 60% humidity on a 12 h light/dark cycle, and provided *ad libitum* access to standard laboratory chow and water. The experimental animal procedures were approved by the Ethics Committee of The Third Hospital Affiliated to Medical University of Zunyi and were performed according to the principles of laboratory animal use and care published by the NIH (NIH publication #85-23, revised in 2011).

We divided the rats into four groups: the middle cerebral artery occlusion (MCAO)-induced ischemic stroke, sham operation, miR-184 adenovirus-injected ischemic stroke, and empty adenovirus vector-injected ischemic stroke groups. The number of surviving animals in each group at the end of the experiment was 14/20 in the ischemic stroke group, 14/14 in the sham operation group, 15/20 in the miR-184 adenovirus-injected ischemic stroke group, and 14/20 in the empty vector-injected ischemic stroke group. A cohort of rats (*n* = 6 from each group) was sacrificed by decapitation after 24 h of reperfusion following MCAO. Brain tissues were carefully removed to assess the infarct volume and miR-184 expression. The remaining rats (*n* = 8–9 in each group) were subjected to analysis of neurological function deficits 1, 3, 7, 14, 21, and 28 days after MCAO using the mNSS system. At the end of the experimental period, the rats were euthanized by CO_2_ asphyxiation.

The sample size was calculated based on the following formula:

n=2.172×(Σ⁢Si2/K)/[Σ⁢(-Xi-X)⁢2/(K-1)]

with Si being the standard deviation estimated for each group, Xi being the mean value of index data estimated for each group, X being the mean value of index data for each group, K being the group number (here, K = 4), and 2.17 being the coefficient when α = 0.05, β = 0.1, and K = 4. The inspection power was 90%.

### Establishment of the Rat Model of Ischemic Stroke

Middle cerebral artery occlusion (MCAO) was used to induce ischemic stroke in rats following Lopez and Vemuganti (2018). Briefly, the rats were anesthetized with 3% isoflurane (AbbVie Inc., United States) and placed on a heating pad, and a midline incision was made in the neck. The right common, external, and internal carotid arteries were carefully separated from the surrounding tissues. The standard and external arteries were ligated. The superficial artery was cut at the distal portion. A silica gel-coated nylon monofilament (0.26 mm) was inserted into the right common carotid artery and advanced into the internal carotid artery to prevent blood flow (Dharap et al., 2009). After 2 h, the monofilament was withdrawn to restore blood circulation, and CT Imaging was performed after 24 h with a micro-CT scanner (Latheta LCT-200, Hitachi Aloka Medical) to assess the outcomes of MCAO. Ischemic stroke modeling was considered successful in rats with low-density regions in the right cerebra because of brain edema caused by the absence of flow (Heiss, 2016). Based on CT images, 2–3 rats from each group were excluded. The sham group underwent the same procedure, but the artery was not ligated and occluded.

### Intracerebroventricular (ICV) Injection

To better determine the effect of miR-184 on cerebral injury after ischemia-reperfusion injury, two groups of rats were subjected to ICV injection of miR-184 adenovirus or empty vector on the right side 2 days before MCAO. In brief, the rats were anesthetized with 3% isoflurane, and a small hole was made in the skull using a hand drill (from bregma: 0.8 mm posterior, -4.8 mm dorsoventral, -1.5 mm lateral) after a sagittal skin incision was made. The coordinates were selected according to Goel et al. (2008) [10]. MiR-184 adenovirus or empty adenovirus (2.5 μl/100 g body weight) (Genechem, Shanghai, China) was microinjected into the lateral ventricles using a Hamilton microsyringe at a rate of 0.5 μl/min. Then, the hole was sealed, and the scalp was sutured.

### Assessment of Neurological Function

Neurological function deficits were assessed 1, 3, 7, 14, 21, and 28 days after MCAO-induced ischemic stroke using the modified neurological severity scoring (mNSS) system by a blinded investigator. The mNSS system assesses movement, sensation, reflex, and balance. Neurological function deficits were graded from 0 to 18 (0, no deficits; 18, maximal deficits) (Germano et al., 1994).

### Assessment of the Infarct Volume

The infarct volume was assessed by 2,3,5-triphenyl tetrazolium chloride (TTC) (Sigma-Aldrich, United States) staining. After the rats were sacrificed by decapitation, the brains were removed and cut into five serial coronal sections (2 mm thick). The slices were then stained with 1% TTC at 37°C for 30 min and fixed in 4% paraformaldehyde. The area of each section was quantified using ImageJ 6.0 software (NIH, Bethesda, MD, United States) following imaging. The cerebral infarct volume was estimated by the equation the infarction area × thickness/2, as described by Wei et al. (2016).

### Quantitative Real-Time PCR (qRT-PCR)

Total RNA was isolated using TRIzol Reagent (Solarbio Co., Beijing, China), and cDNA synthesis was carried out using a cDNA Synthesis Kit (Solarbio Co., Beijing, China). According to the manufacturer’s instructions, PCR amplification was performed with SYBR Green PCR Mix (Solarbio Co., Beijing, China). The sequences of the primers for miR-184 and U6 were as follows: miR-184, forward: 5′-AGT GCA GGG TCC GAG GTA TT-3′, reverse: 5′-CGC GTG GAC GGA GAA CTG AT-3′, stem-loop: 5′-GTC GTA TCC AGT GCA GGG TCC GAG GTA TTC GCA CTG GAT ACG ACA CCC TT; U6, forward: 5′-AGA GAA GAT TAG CAT GGC CCC TG-3′, reverse: 5′-AGT GCA GGG TCC GAG GTA TT-3′, stem-loop: 5′-GTC GTA TCC AGT GCA GGG TCC GAG GTA TTC GCA CTG GAT ACG ACA AAA TA-3′. Relative gene expression was determined by the 2^–ΔΔ*Ct*^ method. Gene expression levels were normalized to the level of U6.

### Cell Culture and Oxygen-Glucose Deprivation

Human SH-SY5Y neuroblastoma cells [American Type Culture Collection (ATCC, Shanghai, China)] were routinely cultured in DMEM/F12 medium (HyClone, Logan, United States) supplemented with 10% fetal bovine serum (Gibco, United States), penicillin (20 U/mL) and streptomycin (20 μg/mL) (Biological Industries, Israel) at 37°C in a humid atmosphere containing 5% CO_2_. Some of these cells was transfected with hsa-miR-184 lentivirus or empty vector or hsa-PPAP2B interference lentivirus or empty vector (Genechem, Shanghai, China) 30 min after polybrene (3 μl/ml) was added to the culture. Subsequently, SH-SY5Y cells with cultured with puromycin to obtain stably transfected cells. After days, cells were transferred to glucose-free DMEM (Logan, United States) in a deoxygenated environment (containing 5% CO_2_ and 95% N_2_) at 37°C for 4 h, and then the cells were incubated in fresh medium containing glucose (4.5 g/L) in 96-well plates at a density of 5 × 10^4^ cells for 24 h in a 5% CO_2_ and 95% O_2_ incubator for oxygen-glucose deprivation/reoxygenation (OGD/R). Control cells were cultured under normal aerobic conditions.

### Cell Viability Assay

Cell viability was determined using the Cell Counting Kit-8 (CCK-8) assay (Beyotime Biotechnology, China). Briefly, after OGD/R, CCK-8 solution (Dojindo, Kumamoto, Japan) was added to each well, and the cells were incubated for 4 more hours at 37°C in a 5% CO_2_ atmosphere according to the manufacturer’s instructions. The absorbance value was measured at 450 nm using a microplate reader (BioTek, United States).

### Cell Apoptosis Assay

Cell apoptosis was assessed using fluorescein isothiocyanate (FITC)-conjugated Annexin V and propidium iodide (P.I.) dual staining. Cells were fixed in 70% ethanol after being washed in phosphate-buffered saline, stained with Annexin V-FITC-propidium iodide (P.I.) (MultiSciences Biotechnology Co., Hangzhou, China), and incubated for 1 h in the dark. then, flow cytometry was performed (3-laser FACSCanto II, B.D. Bioscience, San Jose, CA, United States). The data were analyzed using FlowJo software (Tree Star, United States).

### Dual-Luciferase Reporter Assays

The wild-type 3′ UTR and mutant 3′ UTR of hsa-PPAP2B were amplified and ligated into the pGL3 vector (Promega, United States) containing the luciferase reporter gene. 293T cells (ATCC, Shanghai, China) were cotransfected with hsa-PPAP2B-3′UTR-wt, hsa-PPAP2B-3′UTR-mut, and hsa-miR-184 or its negative control mimic using Lipofectamine 2000 (Invitrogen, China). 293T cells were then incubated in Dulbecco’s modified Eagle’s medium supplemented with 10% fetal bovine serum, 100 U/mL penicillin, and 100 μg/mL streptomycin. After 48 h of culture, a dual-luciferase reporter assay system (Promega, United States) was used to measure luciferase activity.

### Statistical Analysis

The data are expressed as the mean ± *SD*. Comparisons between multiple groups were made by one-way analysis of variance (ANOVA) followed by Tukey’s *post hoc* multiple comparisons test. Neurological scores were analyzed using the Kruskal–Wallis test. *P* < 0.05 was regarded as significant.

## Results

### Expression Levels of MiR-184 in the Brain Are Associated With Brain Injury Following Ischemic Stroke

**FIGURE 1:**
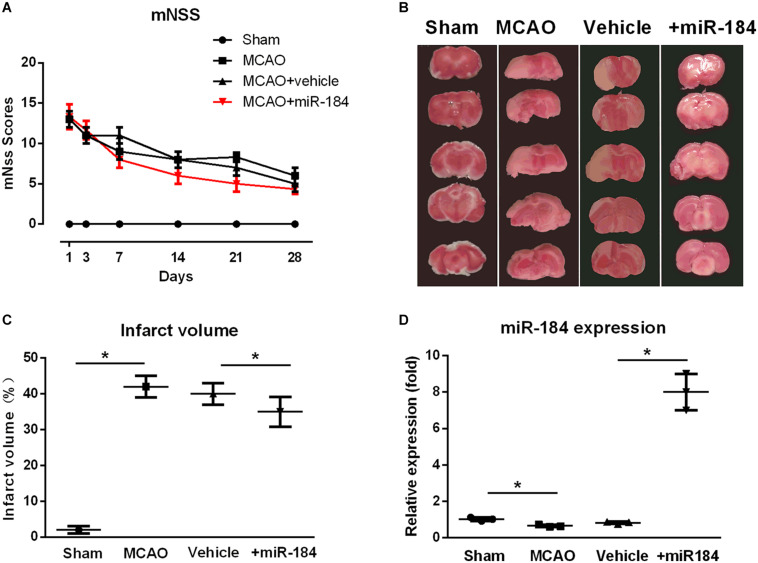
The effect of expression levels of miR-184 in brain on the injured brain following ischemia stroke. Rats were subjected to middle cerebral artery occlusion (MCAO) operation to induce ischemic stroke model with or without intracerebroventricular injection of miR-184 adenovirus (Ad-miR-184). After 24 h following MCAO, a cohort of rats was sacrificed, and brain tissue was taken for TTC staining and qRT-PCR to evaluate the infarct volume and miR-184 expression in brain. Other rats were subjected to analysis of neurological function deficit 1, 3, 7, 14, 21, and 28 day after MCAO operation using mNSS system. **(A)** mNSS scores in various groups (*n* = 8–9). **(B)** TTC-stained brain slice images 24 following ischemia-reperfusion (*n* = 6 in each group). **(C)** Quantification analysis of the infarct volume. **(D)** Relative expression of miR-184 in the brain (*n* = 6 in each group). Values are mean ± *SD*. *p < 0.05 vs. the sham or vehicle.

To evaluate the expression level of miR-184 in the injured brain following ischemic stroke, MCAO was used to establish a rat model of ischemic stroke, and the rats were administered an ICV injection of miR-184 adenovirus or empty vector. We assessed neurological function deficits 1, 3, 7, 14, 21, and 28 days after MCAO using the mNSS system. We found that ischemic stroke rats had higher mNSS scores than sham operation rats, but ischemic stroke rats injected with miR-184 adenovirus had lower mNSS scores than ischemic stroke rats injected with empty vector (Figure 1A).

Some rats of each group were sacrificed after 24 hours, and the brain was collected. Infarct volume was assessed by staining brain tissue with TTC. Non-infarcted brain areas are red, and the infarcted regions are stained white. Consistent with other studies (Bay et al., 2018; etc.), we found that the cerebral infarct volume of ischemic stroke rats was significantly larger than that of sham-operated rats, and they accounted for about 44% of the total brain volume (Figures 1B,C). In contrast, rats injected with adenovirus miR-184 had smaller infarct volumes, accounting for about 35% of total brain volume, than rats injected with an empty vehicle.

Moreover, we also measured the relative expression levels of miR-184 in brain tissue from various groups. qRT-PCR analysis showed that ischemic stroke rats exhibited significantly lower expression of miR-184 than sham rats (*p* < 0.05) and that rats injected with miR-184 adenovirus had higher expression of miR-184 than rats injected with empty vector (*p* < 0.05) (Figure 1D). These results reveal that the expression levels of miR-184 affect nerve injury following ischemic stroke and that downregulation of miR-184 expression after ischemia-reperfusion contributes to brain damage in rats.

### The Effect of MiR-184 Expression Levels on Neuronal Viability and Apoptosis *in vitro*

**FIGURE 2:**
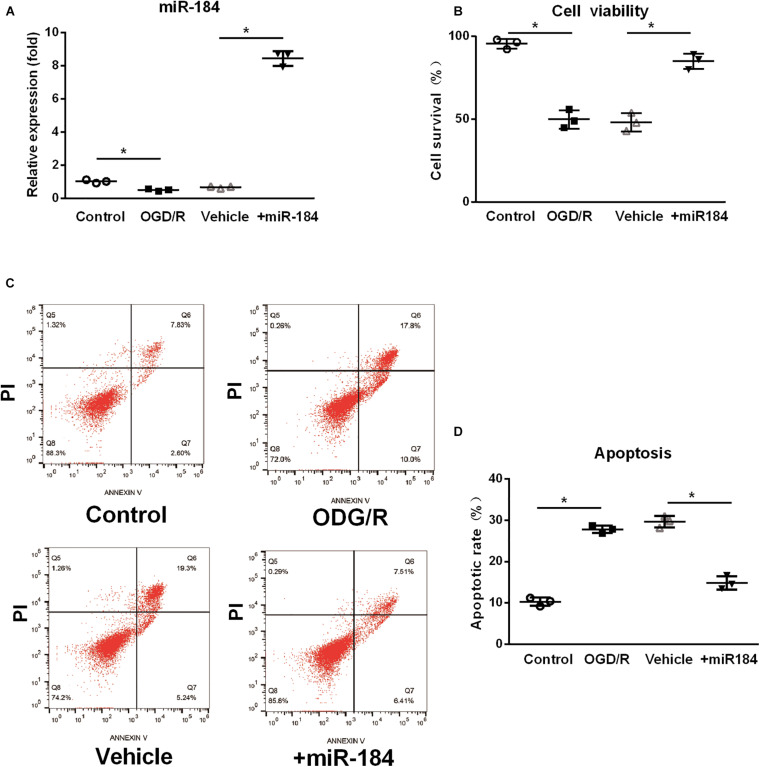
The effect of expression levels of miR-184 on nerve cell viability and apoptosis. Human SH-SY5Y neuroblastoma cells with or without miR-184 lentivirus transduction were challenged with oxygen-glucose deprivation/reoxygenation (OGD/R) to induce ischemia-reperfusion injury *in vitro*. Cell viability, apoptosis and miR-184 expression were detected using CCK-8 assay, flow cytometry and qRT-PCR. **(A)** Relative expression of miR-184 in human SH-SY5Y neuroblastoma cells. **(B)** Cell viability. **(C)** Flow cytometry results. **(D)** Percent of cell apoptosis. Experiments were repeated in triplicate. Values are mean ± *SD*. *p < 0.05 vs. the control or vehicle.

To investigate the primary mechanism by which miR-184 contributes to brain damage following ischemic stroke, we cultured human SH-SY5Y neuroblastoma cells (which exhibit a neuroblast-like morphology), transfected them with or without miR-184 lentivirus, and challenged them with oxygen-glucose deprivation/reoxygenation (OGD/R) to induce ischemic stroke injury *in vitro*. After 24 h, we used qRT-PCR to analyze the relative expression of miR-184 and used the CCK-8 assay and flow cytometry to analyze cell viability and cellular apoptosis. We found that compared to normal aerobic conditions, OGD/R significantly downregulated the expression of miR-184 in SH-SY5Y cells (*p* < 0.05) (Figure 2A), reduced cell viability (p < 0.05) (Figure 2B), and increased cell apoptosis (p < 0.05) (Figures 2C,D). However, miR-184 lentivirus-transfected SH-SY5Y cells exhibited higher expression of miR-184 (*p* < 0.05) (Figure 2A), higher viability (*p* < 0.05) (Figure 2B), and lower apoptosis (*p* < 0.05) (Figures 2C,D) than empty vector-transduced cells following OGD/R challenge. In summary, reduced expression of miR-184 decreases neuronal viability and promotes neuronal apoptosis after ischemia-reperfusion.

### PPAP2B, Target Gene of MiR-184

**FIGURE 3:**
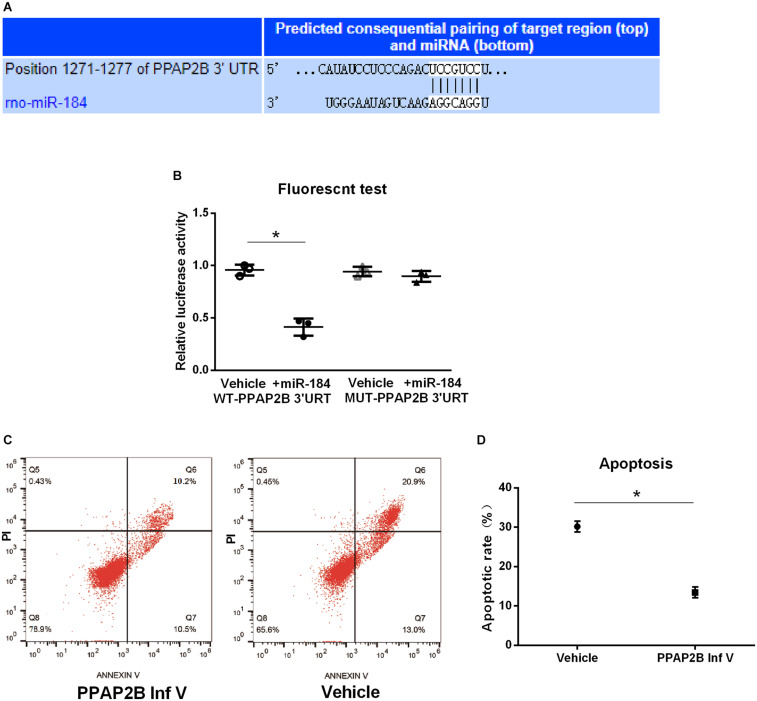
MiR-184 target gene and verification. MiR-184 target gene was predicted using starBase v.2.0 miRNA database (starBase.sysu.edu.cn/), and was verified using dual-luciferase reporter assays. Transduction of SH-SY5Y cells with hsa-PPAP2B interference lentivirus (PPAP2B Inf V) or empty vector and attack with oxygen-glucose deprivation/reoxygenation (OGD/R). Apoptosis was analyzed by flow cytometry to evaluate the effect of the miR-184 target gene on neuronal apoptosis. **(A)** MiR-184 target is phosphatidic acid phosphatase genes type 2B (PPAP2B) mRNA, which can base-pair with miR-184 at its 3’-untranslated region sequences. **(B)** Luciferase activity. **(C)** Flow cytometry results of cell apoptosis. **(D)** Percent of cell apoptosis. Experiments were repeated in triplicate. Values are mean ± *SD*. *p < 0.05 vs. the control or vehicle.

We further predicted the target gene of miR-184 using the starBase v.2.0 miRNA database^1^. We found that miR-184 can target the phosphatidic acid phosphatase type 2B (PPAP2B) gene, which can base-pair with miR-184 at its 3’-untranslated region (Figure 3A).

We also used dual-luciferase reporter assays to verify that the PPAP2B gene is a direct target molecule of miR-184. Hsa-PPAP2B-3′UTR-wt and hsa-PPAP2B-3′UTR-mut plasmids were constructed and cotransfected into 293T cells with hsa-miR-184 or its negative control mimic. Luciferase activity was measured using a dual-luciferase reporter assay system. The results showed that miR-184 lowered the luciferase activity of the hsa-PPAP2B-3′UTR-wt plasmid (*p* < 0.05) but no effect on the hsa-PPAP2B-3′UTR-mut plasmid (Figure 3B), suggesting that miR-184 targets PPAP2B mRNA and inhibits PPAP2B transcription.

Next, we evaluated the effect of suppression of PPAP2B expression on SH-SY5Y cell apoptosis following OGD/R. SH-SY5Y cells were transduced with hsa-PPAP2B interference lentivirus or empty vector and exposed to OGD/R, and after 24 h, cell apoptosis was analyzed by flow cytometry. The results showed that apoptosis was significantly lower (*p* < 0.05) (Figures 3C,D) in hsa-PPAP2B interference lentivirus-transfected SH-SY5Y cells than in empty vector-transfected cells. These results suggest that PPAP2B expression mediates miR-184-induced apoptosis in SH-SY5Y cells following ischemia-reperfusion.

## Discussion

In the current study, we found that MCAO-induced focal cerebral ischemia downregulated the expression of miR-184 and increased the cerebral infarct volume and neurological function deficits. We also showed that ICV injection of miR-184 adenovirus significantly alleviated the cerebral infarct area and neurological function deficits following ischemia stroke in rats. *In vitro* studies demonstrated that OGD/R-induced downregulation of miR-184 expression led to a decrease in viability and an increase in apoptosis in human neuroblastoma SH-SY5Y cells.

In our study, an ischemic stroke rat model was established using the MCAO, which is widely used in stroke-related preclinical studies in rodents. CT imaging was carried out to assess the outcomes of MCAO. Successful establishment of an ischemic stroke model in rats is indicated by the presence of low-density regions in the cerebra. This is because the interruption of blood flow results in brain edema following cerebral artery occlusion. The brain edema area in the ischemic region has a low density upon CT imaging (Heiss, 2016). MCAO commonly results in the loss of various neuronal cells and cerebral infarction (Ferrer and Planas, 2003). The infarct volume depends on multiple factors, including the duration and severity of ischemia, age and sex, and the damage is irreversible by 12 h (Liu and McCullough, 2011).

Along with cerebral infarction, MCAO also results in neurological function deficits, including sensorimotor and cognitive function impairment (DeVries et al., 2001). The extent of neurological function damage can be assessed using behavioral test(s) (DeVries et al., 2001). The mNSS system is often used to evaluate behavioral deficits in animal studies of stroke. The mNSS system can be used to functional deficits in multiple areas (motor, sensory, reflex, and balance) and can be performed within 60 days (Schaar et al., 2010).

MCAO-induced neuronal cell loss occurs through apoptosis, necrosis, autophagocytosis, necroptosis, and pyroptosis and is the core problem in ischemic stroke (Sekerdag et al., 2018). Among the types of cell death that occur after MCAO, apoptosis accounts for the loss of a significant proportion of neuronal cells and prominently occurs in the ischemic penumbra (Radak et al., 2017). Several studies have shown that apoptosis suppression decreases brain damage and behavioral abnormalities and lowers neurological deficit scores after MCAO (Graham and Chen, 2001; Wei et al., 2016; Radak et al., 2017). Postischemic apoptosis is activated by intrinsic mitochondrial signaling pathways or extrinsic apoptotic signaling cascades (Radak et al., 2017).

It has been shown that the expression levels of hundreds of microRNAs are altered following focal cerebral ischemia and reperfusion (Jeyaseelan et al., 2008). Over the past decade, accumulating evidence has identified several brain-enriched miRNAs as critical mediators in the multifaceted cascade of focal cerebral ischemia pathology, including excitotoxicity, oxidative stress, inflammatory reaction, blood-brain barrier disruption, edema after stroke, neuronal damage or death, neurogenesis, and angiogenesis (Li et al., 2018). Some microRNAs are implicated in neuronal damage or death; for example, alterations in the expression levels of miR-7a-5p, miR-9, miR-21, miR-25, miR-29b, miR-181, and miR-497 are involved in neuron apoptosis, and the miR-30 family, miR-26b, and miR-207 affect autophagocytosis in the postischemic brain and neuronal cultures under OGD/R conditions (Rink and Khanna, 2011; Wei et al., 2016; Kim et al., 2018; Li et al., 2018). Supporting the notion that microRNAs impact apoptosis, in the current study, we demonstrated that downregulation of miR-184 expression resulted in apoptosis in human neuroblastoma SH-SY5Y cells following OGD/R.

In the current study, it was confirmed that miR-184 contributes to neuronal apoptosis following ischemia and reperfusion in human neuroblastoma SH-SY5Y cells cultured under OGD/R conditions. Human neuroblastoma SH-SY5Y cells are neuroblast-like cells that are most commonly used *in vitro* cerebral ischemia-related research (Kovalevich and Langford, 2013; Liu et al., 2018). OGD/R is the most widely used *in vitro* model of ischemic stroke and can be used to model ischemia-reperfusion induced by combined oxygen and glucose deprivation (Sommer, 2017). Typically, 1 h exposure to OGD/R is sufficient to cause widespread neuronal death (Sommer, 2017). Consistent with other studies (Sommer, 2017), we found that OGD/R significantly increased the apoptosis of SH-SY5Y cells. However, overexpression of miR-184 reduced SH-SY5Y cell apoptosis.

In our study, we also found that PPAP2B is a direct target gene of miR-184. PPAP2B encodes phosphatidic acid phosphatase (also named lipid phosphate phosphatase 3), which catalyzes the dephosphorylation of various lipid phosphates into diacylglycerol and is implicated in the physiological processes of cell proliferation, differentiation, and apoptosis (Ishikawa et al., 2000; Long et al., 2005). It is well-known that diacylglycerol is an essential activator of protein kinase C. Some evidence has shown that protein kinase C plays a crucial role in mediating cerebral reperfusion injury (Zhao et al., 2016). In particular, protein kinase C delta isozyme expression is specifically upregulated and activated after ischemia and reperfusion in the perifocal cortex and has been reported to mediate apoptotic processes (Bright and Mochly-Rosen, 2005). It has been shown that protein kinase C delta isozyme can lead to mitochondrial dysfunction and the release of apoptogenic factors in the ischemic heart (Murriel et al., 2004). Identical findings have also been demonstrated in a cerebral ischemia model (Bright et al., 2004). Therefore, in our study, miR-184 downregulation-induced SH-SY5Y apoptosis may have been mediated through protein kinase C delta isozyme via phosphatidic acid phosphatase encoded by PPAP2B. Indeed, lipid phosphate phosphatase 3 has been shown to regulate the survival of other cells. For instance, the overexpression of the PPAP2B gene significantly increases apoptosis of human primary aortic endothelial cells, and PPAP2B silencing by siRNA reduces cell apoptosis (Touat-Hamici et al., 2016). Similarly, PPAP2B-overexpressing HEK-293 cells undergo apoptosis after serum deprivation (Long et al., 2005).

MiR-184 has emerged as a highly enriched microRNA in the mammalian brain (Nomura et al., 2008). However, the exact role of miR-184 in the brain is not yet well known. Several studies have shown that miR-184 plays roles in physiological and pathophysiological processes in the brain. For example, miR-184 can promote oligodendrocyte differentiation, control the proliferation and differentiation of adult neural stem/progenitor cells, and suppress glioma proliferation, migration, and invasion (Liu et al., 2010; Cheng et al., 2015; Afrang et al., 2019), and suppression of miR-184 expression results in neuronal death after seizures in mice (McKiernan et al., 2012). Our previous study demonstrated that miR-184 expression is significantly downregulated in the rat brain and in cultured cells following ischemia and reperfusion, which modulates post-OGD/R angiogenesis *in vitro* (Wang et al., 2020). The results of the current study also revealed that downregulation of miR-184 expression contributes to neuronal cell damage and death in rats following ischemic stroke and induces SH-SY5Y cell apoptosis under OGD/R conditions. However, it is still not clear how downregulation of miR-184 expression is triggered *in vivo* and *in vitro*. A decrease in miR-184 levels appears to induce the death of SH-SY5Y cells. It has been reported that antagomir-mediated inhibition of miR-184 also results in neuronal death in the hippocampal CA1 area in mice following seizures (McKiernan et al., 2012).

Biological validation of the role of microRNAs in ischemic stroke is providing insight into the complex molecular mechanisms underlying ischemic stroke and new therapeutic strategies for ischemic stroke, such as targeting microRNAs to modulate discordant gene expression, which may represent the future of gene therapy for ischemic stroke. There have been preclinical studies on the potential of targeting microRNAs to protect against brain damage following ischemic stroke. A miR-15a/16-1 antagomir, miR-93 antagomir, miR-106b-5p antagomir, miR-181 antagomir, miR-383 antagomir, miR-497 antagomir, miR-122 mimic, and miR-1906 mimic have been proven have therapeutic potential in protecting against cerebral ischemia and reperfusion injury (Yin et al., 2010; Khoshnam et al., 2017b; Wang et al., 2018). Our study on miR-184 in ischemic brain injury suggests that targeting brain-specific miR-184 may be a promising therapeutic option for stroke recovery.

Our results revealed the critical role of miR-184 in cerebral ischemia and reperfusion injury and demonstrated that the attenuation of miR-184 following ischemia and reperfusion contributes to neuronal damage in rats and increases SH-SY5Y cell apoptosis through direct targeting of PPAP2B mRNA. Our study may provide a promising therapeutic option for stroke recovery.

## Data Availability Statement

The raw data supporting the conclusions of this article will be made available by the authors, without undue reservation.

## Ethics Statement

The animal study was reviewed and approved by the Ethics Committee of The Third Hospital Affiliated to Medical University of Zunyi.

## Author Contributions

HY, YZ, and HC searched literatures, performed the experiments, and analyzed the data. HY, YZ, YL, and FO wrote the manuscript. LC and DL designed the experiments and revised the manuscript. All authors have approved the final article and agreed to be accountable for the content of the work.

## Conflict of Interest

The authors declare that the research was conducted in the absence of any commercial or financial relationships that could be construed as a potential conflict of interest.

## Footnotes

1 starBase.sysu.edu.cn/
