# Supplementary figures and images for: Mir-184 Contributes to Brain Injury Through Targeting PPAP2B Following Ischemic Stroke in Male Rats
Source: Front Mol Neurosci. 2021 Mar 23;14:613887. doi: 10.3389/fnmol.2021.613887 (PMC8021718; doi:10.3389/fnmol.2021.613887)

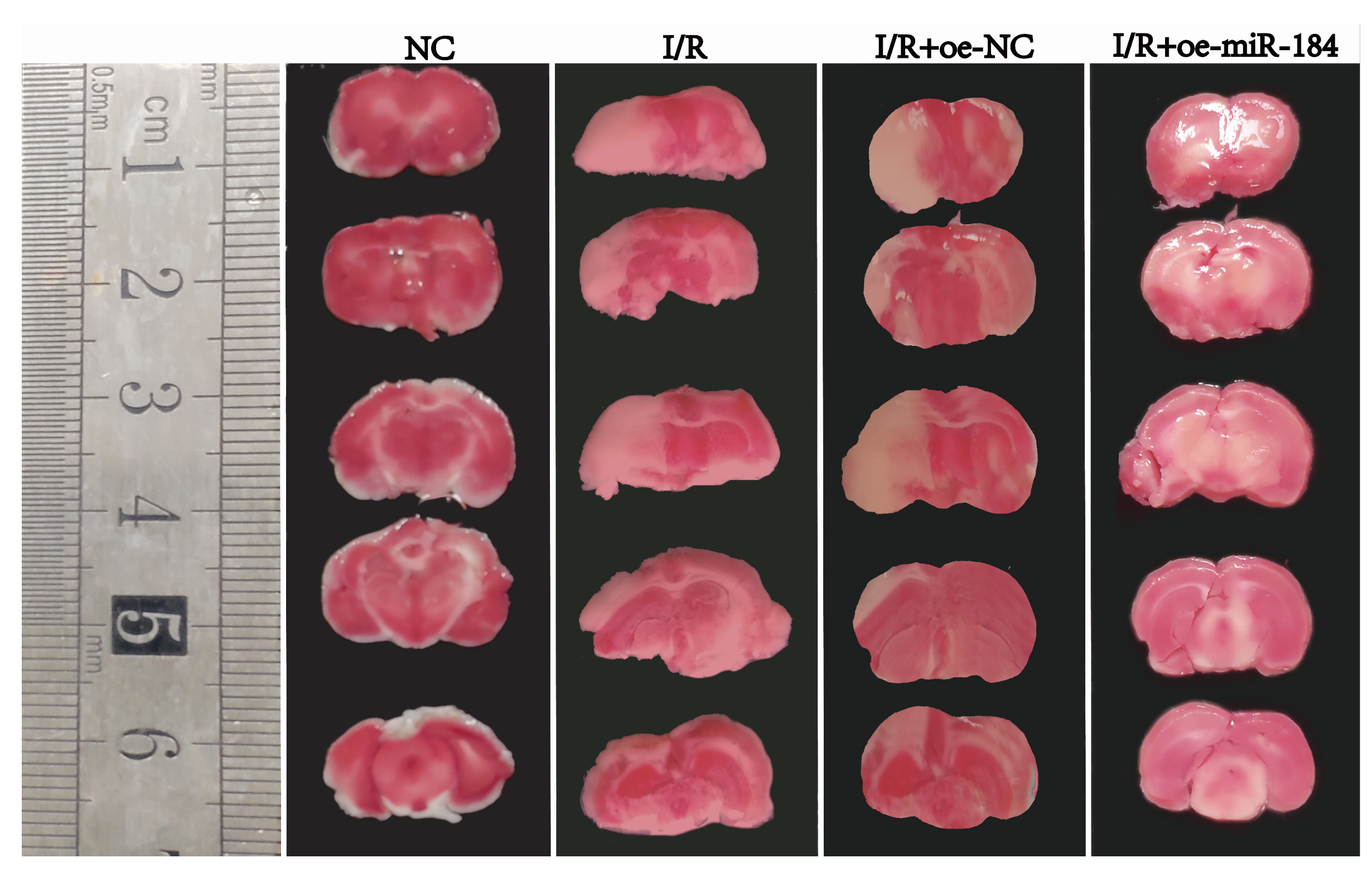

Supplement: Supplementary file 1 [file Image_1.PNG]
